# Supplementary material for: A multivesicular body-like organelle mediates stimulus-regulated trafficking of olfactory ciliary transduction proteins
Source: Nat Commun. 2022 Nov 12;13:6889. doi: 10.1038/s41467-022-34604-y (PMC9653401; doi:10.1038/s41467-022-34604-y)
Supplement: Supplementary file 1 — Supplementary Information. [file 41467_2022_34604_MOESM1_ESM.pdf]

Supplementary Figures

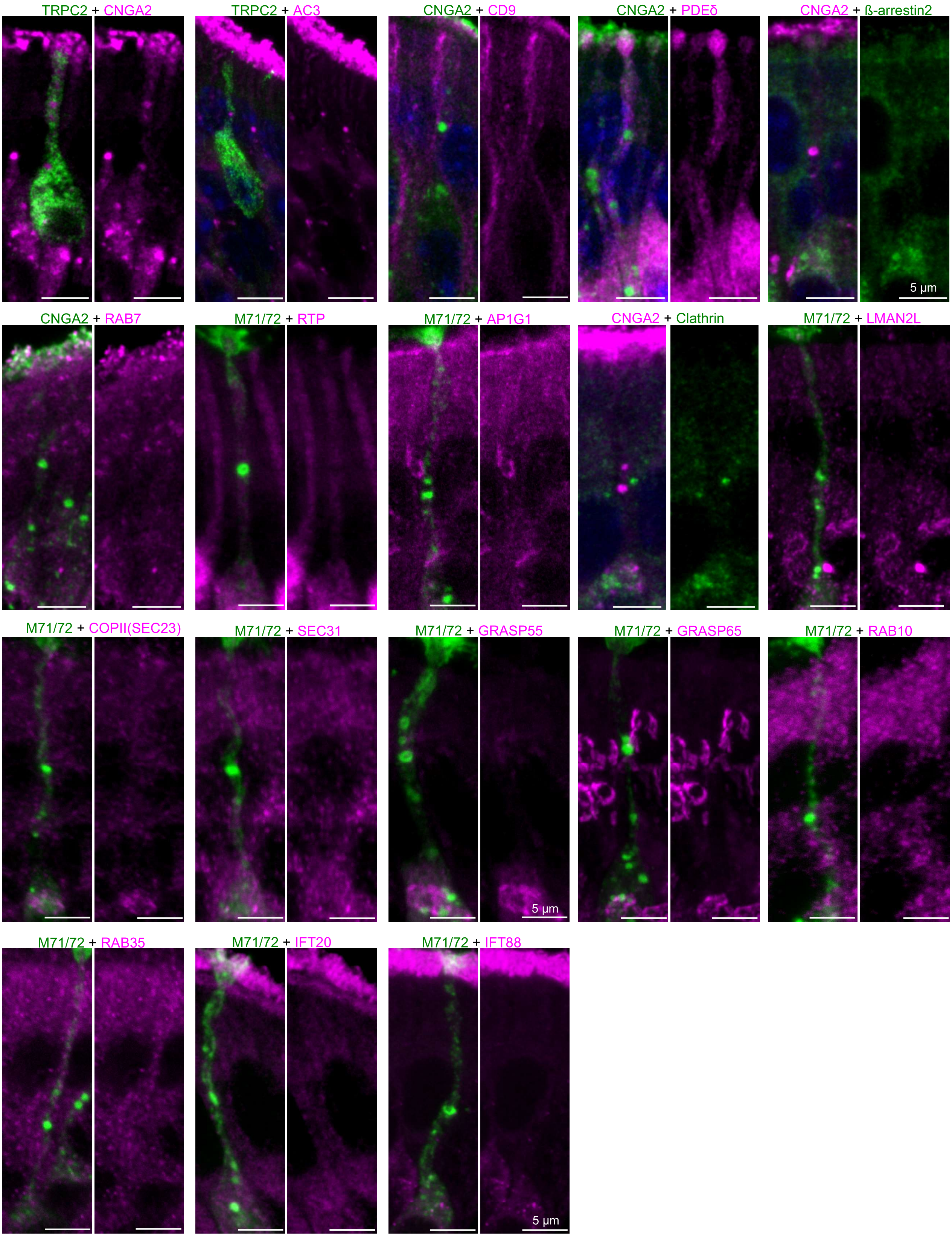

Supplementary Fig. 1: Represntative images of double immunohistochemistry showing that $AC3^{+}$, $CNGA2^{+}$ and $M71/72^{+}$ dendritic puncta did not colocalize with TRPC2, CD9, PDEδ, β-arrestin2, RAB7, RTP, AP1G1, Clathrin, LMAN2L, COPII (SEC23), SEC31, GRASP55, GRASP65, RAB10, RAB35, IFT20, or IFT88. Images for M71/72 (green channel) are from Fig. 3A, which illustrate the distribution of MVTs in the dendrite. 2-3 mice were analyzed per experiment.

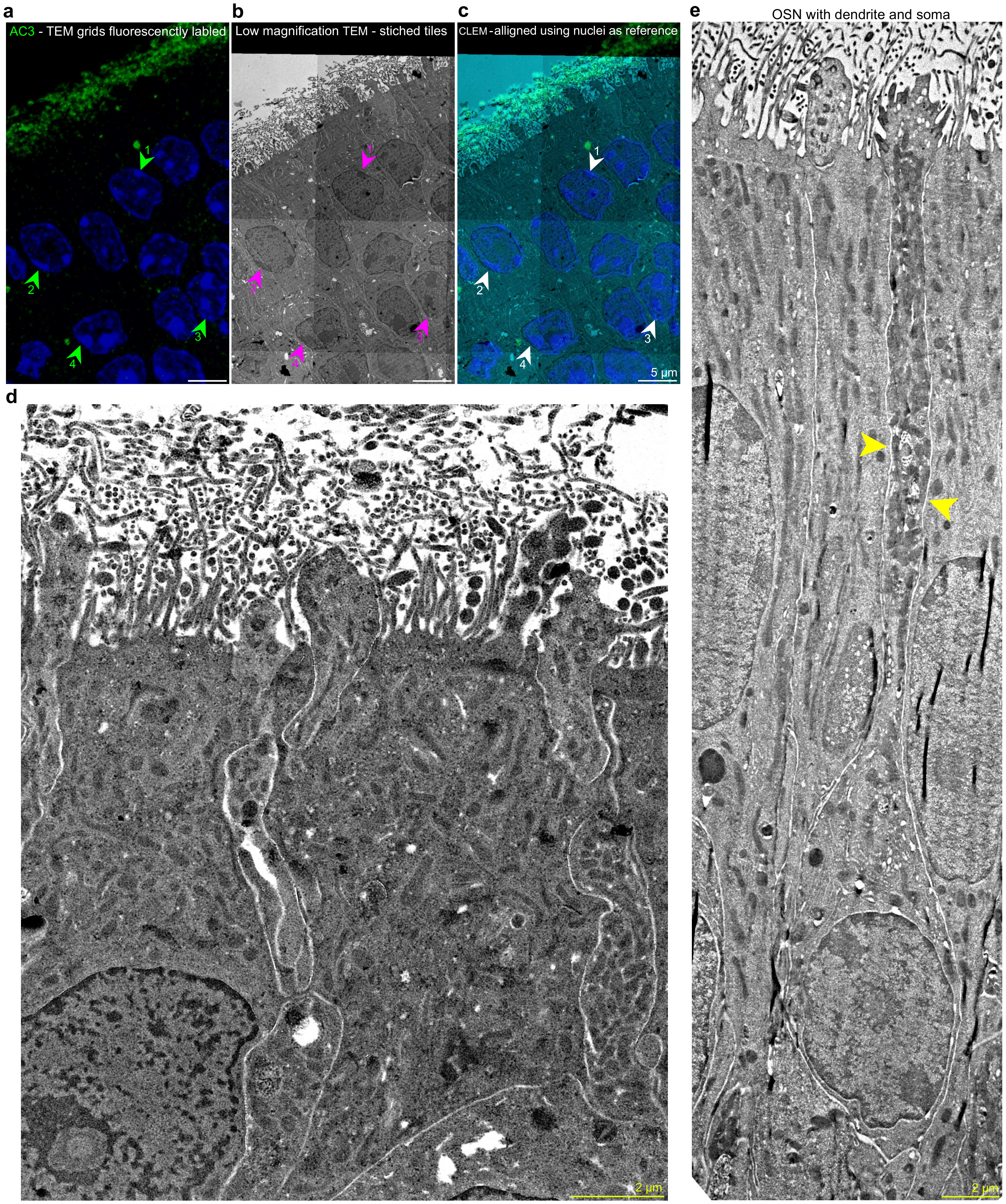

Supplementary Fig. 2: Alignment of CLEM images and TEM images in low magnification. a, A confocal scan of a TEM grid with AC3 immunofluorescence and Hoechst stained nuclei is shown. **b,** A TEM grid with stitched tiles is shown. **c,** Shown is an alignment of the images shown in **a** and **b**. Reference points (arrowheads) used for the alignment were electron dense nuclear areas (heterochromatin) in the TEM image and intensely stained Hoechst immunofluorescence in the confocal image. **d,** The TEM image from the CLEM analysis in Fig. 1f without fluorescence overlay and at a lower magnification is shown. **e,** A low magnification TEM scan of an OSN with two MVTs (arrowheads) is shown. 4 mice were analyzed per experiment.

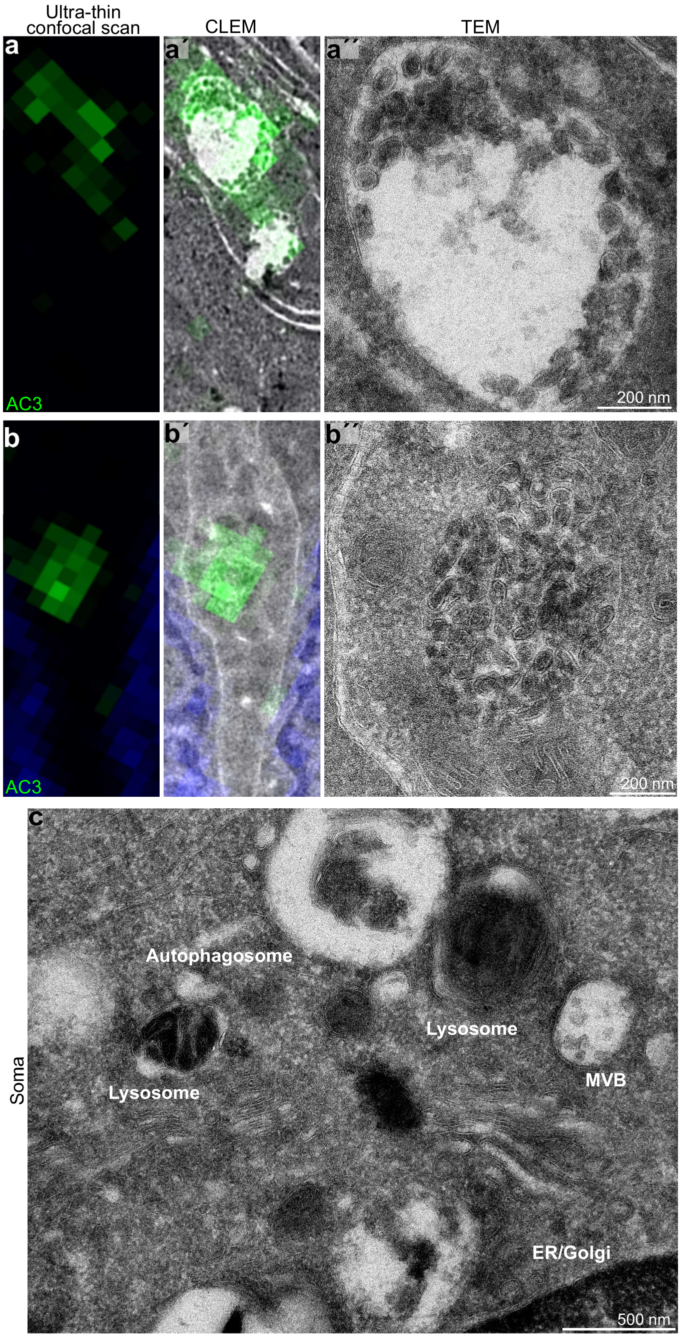

Supplementary Fig. 3: MVB-like organelles are in dendrites and soma. Lysosomes and autophagosomes are restricted to soma. Representative confocal (**a**, **b**), CLEM (**a´**, **b´**) and TEM (**a´´**, **b´´**) images showing AC3^{+} MVB-like organelles in dendrites. **c,** Representative TEM image of a soma showing organelles that resemble lysosomes, autophagosomes, MVBs, the ER and the Golgi apparatus. 3-4 mice were analyzed per experiment.

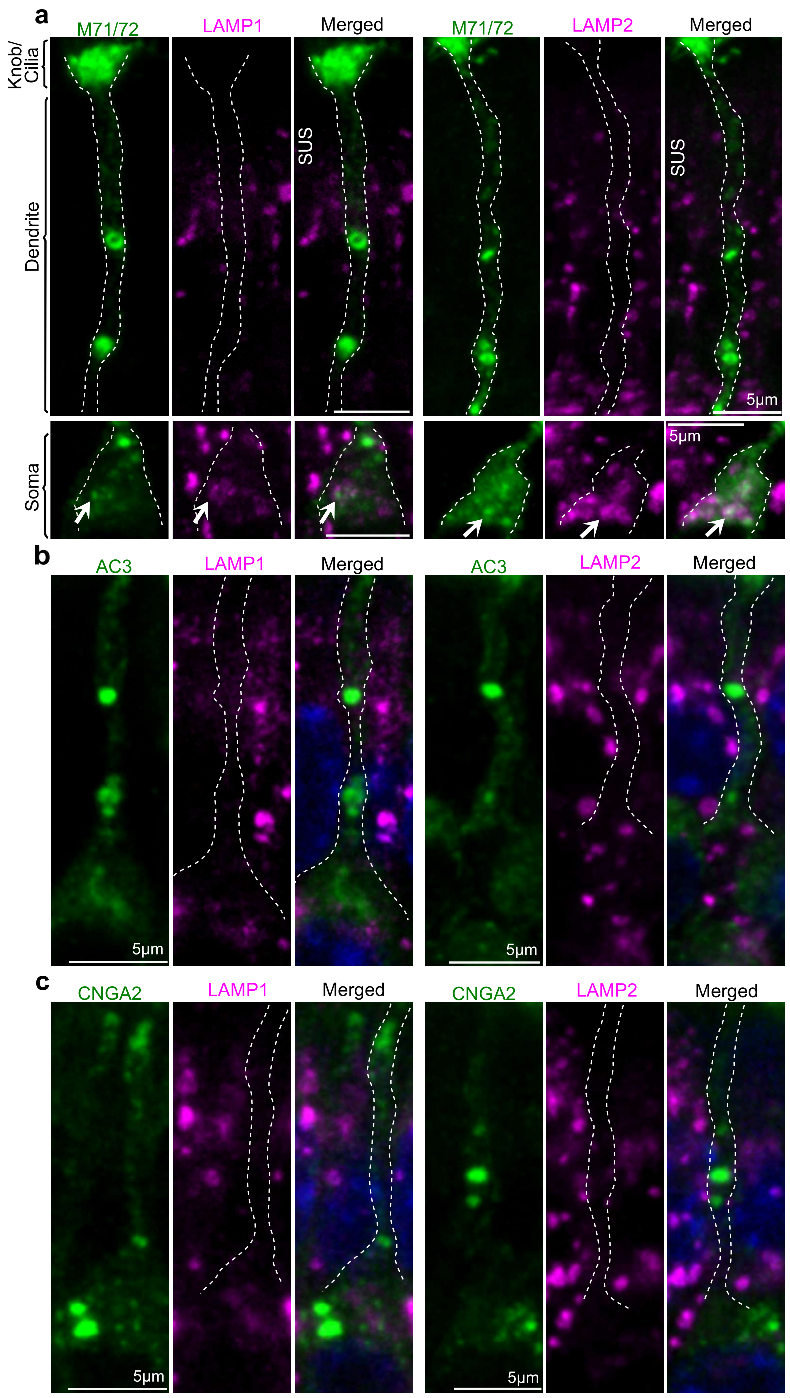

Supplementary Fig. 4: LAMP1/2 expression and lysosomes in the soma but not in dendrites. a, Representative images of double immunohistochemistry for M71/72/LAMP1 and M71/72/LAMP2 in the dendrites and somas of OSNs (outlined) and flanking sustentacular cells (SUS). M71/72$^+$ puncta (green) are in OSN dendrites and somas. LAMP1$^+$ and LAMP2$^+$ puncta (magenta) were present in sustentacular cells and in OSN somas, but not in dendrites. A few M71/72$^+$ puncta in the soma colocalized with LAMP1/2 (arrows). **b,** Images of double staining for AC3/LAMP1 and AC3/LAMP2, are shown. **c,** CNGA2/LAMP1 and CNGA2/LAMP2 double stainings showing LAMP1 and LAMP2 expression in soma but not in dendrites. 3 mice were analyzed per experiment.

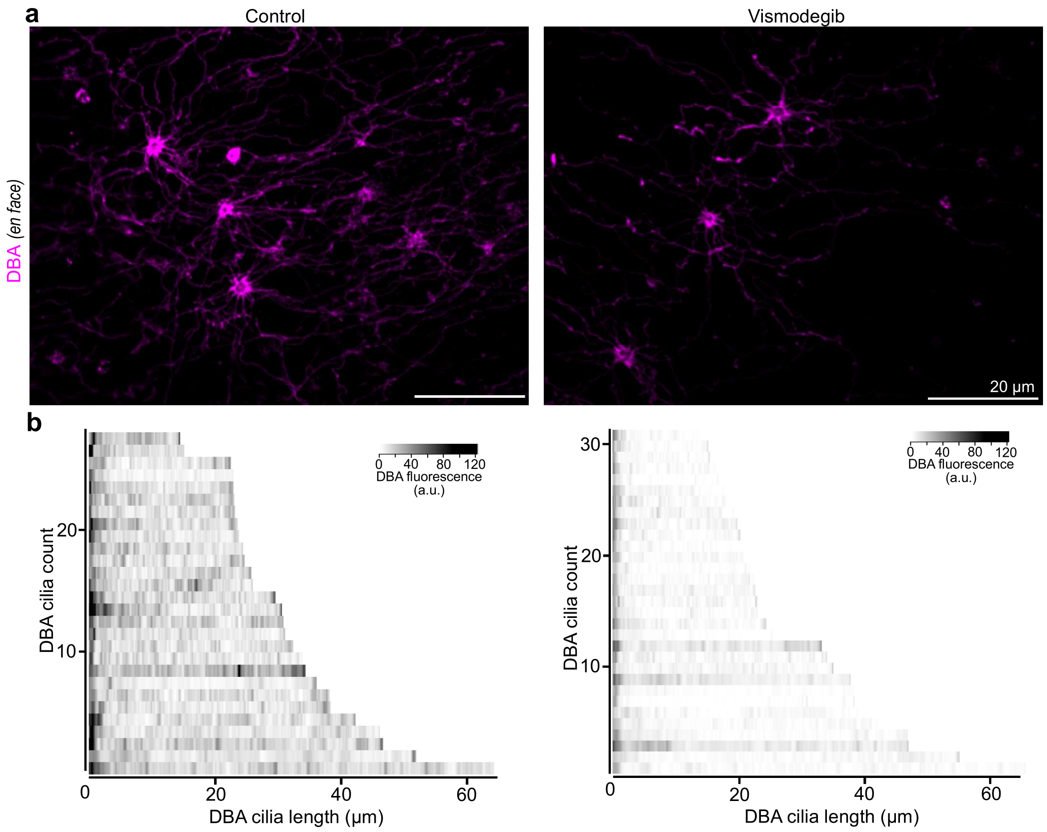

Supplementary Fig. 5: Vismodegib inhibits the localization of ORs to cilia in OSNs. a, En face preparation of OE tissue showing DBA fluorescence in the dendritic knobs and cilia. **b,** Heatmaps of the DBA fluorescence intensity in 28 cilia from control mice and 31 cilia from vismodegib-treated mice (n = 3).

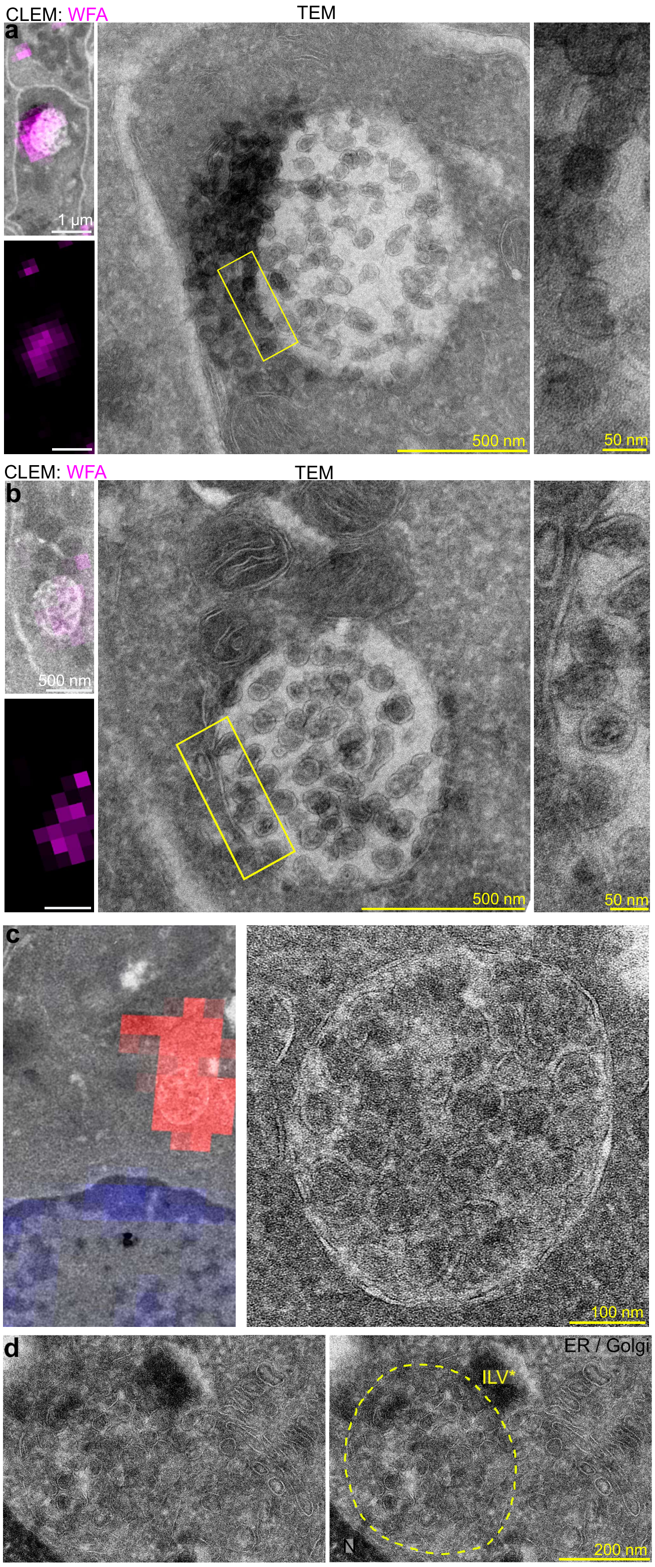

Supplementary Fig. 6: Presence of ILV* clusters in the distal dendritic segment and soma. a, An MVT with a disintegrated limiting membrane and clusters of WFA$^+$ ILVs* that appear to burst out from the MVT into the cytosol of the dendrite is shown. **b,** An MVT with an intact limiting membrane and WFA$^+$ ILVs is shown. High-magnification TEM images of the regions delimited by the yellow rectangles are shown on the right in **a-b**. **c,** Shown is a WFA$^+$ MVB-like organelle in the soma close to the nucleus (blue). **d,** Shown is a putative ILV* cluster close to ER/Golgi. The yellow dotted circle indicates the border between the ILV* cluster and cytoplasm. 3 mice were analyzed per experiment.

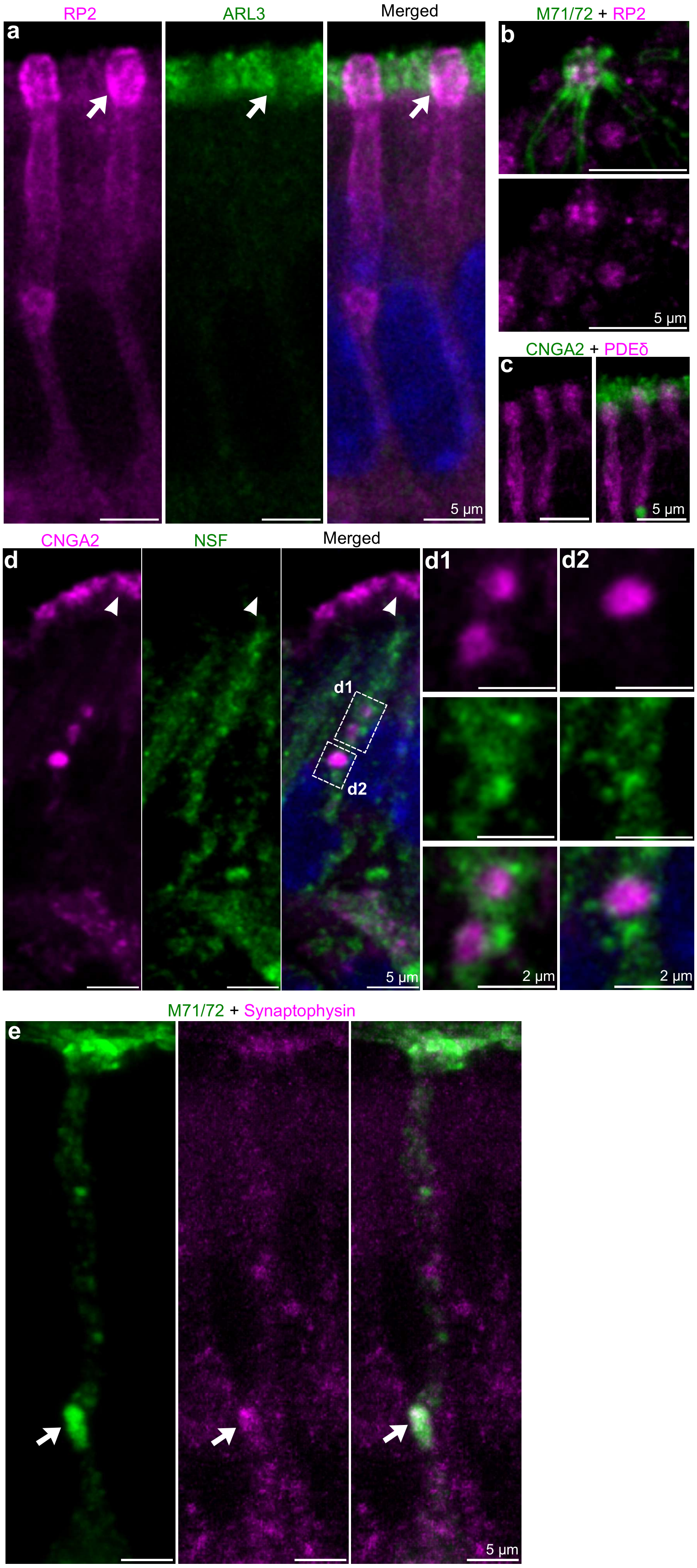

Supplementary Fig. 7: Localization of ARL3, PDEδ, NSF and synaptophysin. a, Representative double immunohistochemistry image showing colocalization of RP2 (magenta)/ARL3 (green) in the dendritic knob (arrow). **b,** En face image of the OE showing overlapping M71/72 and RP2 immunofluorescence at the ciliary bases in the dendritic knob. **c,** Overlapping PDEδ (magenta) and CNGA2 (green) immunofluorescence within the dendritic knob is shown. **d,** Representative double immunohistochemistry image for CNGA2 and NSF showing that NSF fluorescence partly overlapped with MVTs, which are outlined and shown at high magnification in **d1** and **d2**. **e,** Representative double immunohistochemistry image for M71/72/synaptophysin showing colocalization in an MVT (arrow). 3 mice were analyzed per experiment.

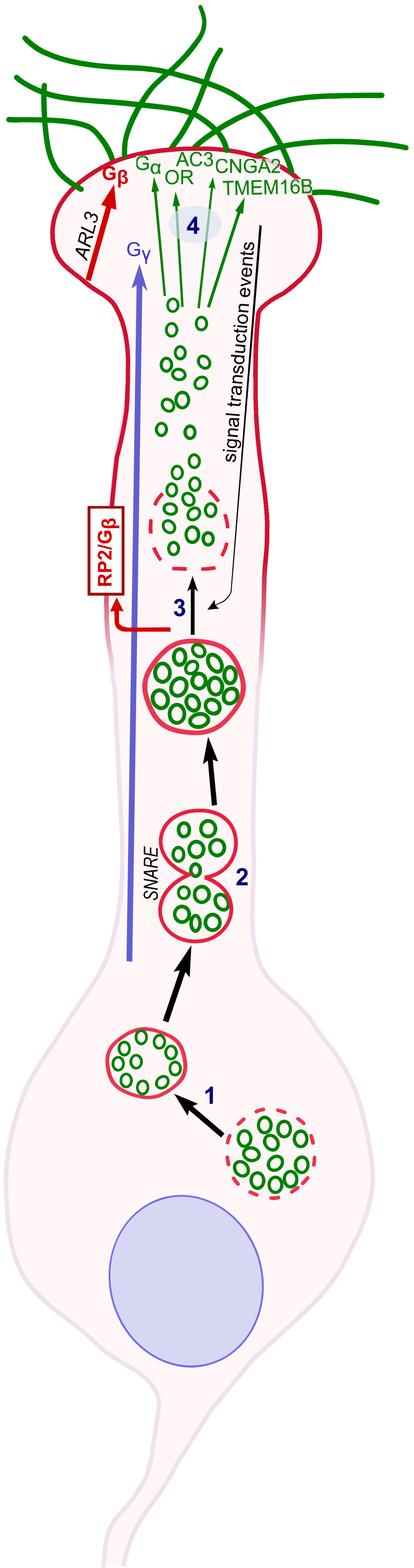

Supplementary Fig. 8: Schematic representation of the proposed MVT pathway. Depicted are the proposed stages of the MVT pathway, which are as follows: **1.** MVT biogenesis in the soma; **2.** Possible homotypic vesicle fusion mediated by SNARE complex proteins; **3.** Odorant-induced disintegration of the limiting membrane and translocation of RP2 and Gβ1 (Gβ) to the plasma membrane; and **4.** Assembly and ciliary targeting of olfactory transduction proteins at the ciliary bases in the dendritic knob, where RP2- and ARL3-regulated ciliary targeting takes place. The G protein subunit Gβ1 (Gβ) is associated with RP2 in the limiting and plasma membrane (red) and Gγ13 (Gγ) reaches the dendritic knob in vesicles (blue) distinct from the MVT. Gαolf (Gα), OR, AC3, CNGA2 and TMEM16B reach the dendritic knob in ILVs (green).

Supplementary table 1: Details of primary and secondary antibodies.

Antibody	Company	Raised in	Dilution	Catalogue	RRID	Specificity reporting citations / own evaluation
CNGA2	Santa Cruz Biotechnology	Goat	1:50	sc-13700	AB_2081833	doi.org/10.1002/dneu.22159
AP1G1	Proteintech Group	Rabbit	1:500	13258-1-AP	AB_2058209	doi.org/10.3389/fcell.2019.00181
ARL3	Proteintech Group	Rabbit	1:100	10961-1-AP	AB_2274220	doi.org/10.1038/ncomms6295
Beta Arrestin 2	Proteintech Group	Rabbit	1:100	10171-1-AP	AB_10644158	doi.org/10.1038/cddis.2016.89
CHMP1A	Proteintech Group	Rabbit	1:500	15761-1-AP	AB_2229399	doi.org/10.1016/j.celrep.2018.06.100
CHMP4B	Thermo Fisher Scientific	Rabbit	1:400	PA5-100092	AB_2815622	Manufacturer's confirmation: with blocking peptides
COPII	Thermo Fisher Scientific	Rabbit	1:500	PA1-069A	AB_2301581	doi.org/10.1016/j.jbc.2021.101536
GRASP55	Proteintech Group	Rabbit	1:200	10598-1-AP	AB_2113473	doi.org/10.4049/jimmunol.1901124<br>doi.org/10.1083/jcb.200907132
GNG13	Atlas Antibodies	Rabbit	1:500	HPA046272	AB_10960062	doi.org/10.1016/j.prp.2020.153143<br>doi.org/10.1038/s41586-018-0393-7
GRASP65	Thermo Fisher Scientific	Rabbit	1:500	PA3-910	AB_2113207	doi.org/10.1038/s41598-019-53124-2<br>doi.org/10.1007/s10495-019-01579-z
HGS	GeneTex	Rabbit	1:400	GTX101718	AB_2037164	doi.org/10.1371/journal.ppat.1005123
IFT20	Proteintech Group	Rabbit	1:150	13615-1-AP	AB_2280001	doi.org/10.1111/cas.13970<br>doi.org/10.1038/s41418-019-0357-y
IFT88	Proteintech Group	Rabbit	1:150	13967-1-AP	AB_2121979	doi.org/10.1038/s41388-018-0211-6
LMAN2L	Proteintech Group	Rabbit	1:100	17877-1-AP	AB_2878459	Manufacturer's confirmation with siRNA
LAMP1	GeneTex	Rat	1:400	GTX42501	AB_11162924	doi.org/10.1016/S0021-9258(18)34878-6
LAMP2	Thermo Fisher Scientific	Rat	1:400	14-1072-82	AB_657560	doi.org/10.1016/j.bbrep.2016.01.010
NSF	Novus Biologicals	Rabbit	1:500	NBP1-87035	AB_11010816	doi.org/10.1007/s12031-014-0231-9
S100A5	Thermo Fisher Scientific	Mouse	1:500	MA5-29536	AB_2785384	validated in author´s laboratory by unilateral naris occlusion
RAB10	Proteintech Group	Rabbit	1:200	11808-1-AP	AB_2173442	doi.org/10.1074/jbc.RA118.007318
RAB35	Thermo Fisher Scientific	Rabbit	1:200	PA5-31674	AB_2549147	Manufacturer's confirmation: with blocking peptides

RAB7	Proteintech Group	Rabbit	1:400	55469-1-AP	AB_11182831	doi.org/10.1038/s41419-021-03670-3
RP2	Proteintech Group	Rabbit	1:500	14151-1-AP	AB_11182501	doi.org/10.1016/j.stemcr.2020.05.007
RTP1	Proteintech Group	Rabbit	1:500	18973-1-AP	AB_10550546	Manufacturer's confirmation
SEC31	Proteintech Group	Rabbit	1:500	17913-1-AP	AB_2186378	doi.org/10.1038/s41586-021-04109-7
Synaptophysin	Synaptic Systems	Mouse	1:50	101 011	AB_887824	doi.org/10.1046/j.1460-9568.1999.00542.x
STAM1	Proteintech Group	Rabbit	1:300	12434-1-AP	AB_2199965	doi.org/10.1074/jbc.M116.757138
TSG101	Novus Biologicals	Rabbit	1:100	NBP2-67884	AB_2922669	doi.org/10.1111/jcmm.16002
VPS4A	LSBio	Rabbit	1:500	LS-C346226	AB_2922670	Manufacturer's confirmation: using WB and IHC
Golfα (GNAL)	Santa Cruz Biotechnology	Mouse	1:200	sc-55545	AB_831819	doi.org/10.1038/s41467-017-02661-3 doi.org/10.1093/schbul/sbv129
TRPC2	BiCell scientific	Rat	1:200	11012	AB_2922678	Manufacturer's confirmation: by IHC and own lab confirmation in VNO and OE
M71/M72	Lomvardas S et al. 2006	Guinea Pig	1:2000			Lomvardas S et al. 2006
AC3	Santa Cruz Biotechnology	Rabbit	1:100	sc-588	AB_630839	doi.org/10.1016/S0896-6273(00)00060-X
S100A5	Schäfer BW et al. 2000	Rabbit	1:2000	62FAC1		Schäfer BW et al. 2000
Gβ1	Santa Cruz Biotechnology	Mouse	1:500	Sc-166123	AB_2109632	doi.org/10.7554/eLife.54298 doi.org/10.3892/etm.2022.11450
CD9	BD Bioscience	Rat	1:500	553758	AB_395032	doi.org/10.4049/jimmunol.166.5.3256
PDEδ	Atlas Antibodies	Rabbit	1:500	HPA037433	AB_2675475	Manufacturer's confirmation: using WB and IHC

Alexa 488-conjugated anti-guinea pig	Jackson ImmunoResearch Europe	Donkey	1:500	706–545-148	AB_2340472
Alexa 488-conjugated anti-rabbit	Life Technologies	Donkey	1:500	A-21206	AB_2535792
Alexa 546-conjugated anti-rabbit	Life Technologies	Donkey	1:500	A-10040	AB_2534016
Alexa 546-conjugated anti-goat	Life Technologies	Donkey	1:500	A-11056	AB_2534103
Dylight 488-conjugated anti-mouse	Agrisera AB	Donkey	1:500	AS101201	AB_10750102
Alexa 546-conjugated anti-rat	Life Technologies	Donkey	1:500	A-21208	AB_2535794
Cy™3 conjugated anti-guinea Pig	Jackson ImmunoResearch Europe	Donkey	1:500	706-165-148	AB_2340460
