# Supplementary material for: A multivesicular body-like organelle mediates stimulus-regulated trafficking of olfactory ciliary transduction proteins
Source: Nat Commun. 2022 Nov 12;13:6889. doi: 10.1038/s41467-022-34604-y (PMC9653401; doi:10.1038/s41467-022-34604-y)
Supplement: Supplementary file 3 — Reporting Summary [file 41467_2022_34604_MOESM3_ESM.pdf]

nature portfolio

Corresponding author(s): Staffan Bohm

Last updated by author(s): Oct 17, 2022

Reporting Summary

Nature Portfolio wishes to improve the reproducibility of the work that we publish. This form provides structure for consistency and transparency in reporting. For further information on Nature Portfolio policies, see our Editorial Policies and the Editorial Policy Checklist.

Statistics

For all statistical analyses, confirm that the following items are present in the figure legend, table legend, main text, or Methods section.

n/a	Confirmed	
☐	☒	The exact sample size (*n*) for each experimental group/condition, given as a discrete number and unit of measurement
☐	☒	A statement on whether measurements were taken from distinct samples or whether the same sample was measured repeatedly
☐	☒	The statistical test(s) used AND whether they are one- or two-sided<br>*Only common tests should be described solely by name; describe more complex techniques in the Methods section.*
☒	☐	A description of all covariates tested
☒	☐	A description of any assumptions or corrections, such as tests of normality and adjustment for multiple comparisons
☐	☒	A full description of the statistical parameters including central tendency (e.g. means) or other basic estimates (e.g. regression coefficient) AND variation (e.g. standard deviation) or associated estimates of uncertainty (e.g. confidence intervals)
☐	☒	For null hypothesis testing, the test statistic (e.g. *F*, *t*, *r*) with confidence intervals, effect sizes, degrees of freedom and *P* value noted<br>*Give P values as exact values whenever suitable.*
☒	☐	For Bayesian analysis, information on the choice of priors and Markov chain Monte Carlo settings
☒	☐	For hierarchical and complex designs, identification of the appropriate level for tests and full reporting of outcomes
☒	☐	Estimates of effect sizes (e.g. Cohen's *d*, Pearson's *r*), indicating how they were calculated

Our web collection on statistics for biologists contains articles on many of the points above.

Software and code

Policy information about availability of computer code

Data collection	The following softwares were used to collect the data:<br>-Leica Application Suite X 3.5.7.23225<br>-Velox 2.14.2.40 Software<br>-TIA 5.0 Software<br>-Maps 3.3 application software
Data analysis	The following softwares were used to analyze the data:<br>- FIJI image processing software (NIH) - ImageJ 1.53c<br>- Maps 3.3 application software<br>- 3D Visualization-Assisted Analysis Suite (Vaa3D) v 3.601<br>- Microsoft Office Excel<br>- R version 4.0.5

For manuscripts utilizing custom algorithms or software that are central to the research but not yet described in published literature, software must be made available to editors and reviewers. We strongly encourage code deposition in a community repository (e.g. GitHub). See the Nature Portfolio guidelines for submitting code & software for further information.

Data

Policy information about availability of data

All manuscripts must include a data availability statement. This statement should provide the following information, where applicable:

- Accession codes, unique identifiers, or web links for publicly available datasets
- A description of any restrictions on data availability
- For clinical datasets or third party data, please ensure that the statement adheres to our policy

Data that support the findings of this study is present in the paper, supplementary information, Source Data and the Zenodo database (10.5281/zenodo.7194768).

Human research participants

Policy information about studies involving human research participants and Sex and Gender in Research.

Reporting on sex and gender	N/A
Population characteristics	N/A
Recruitment	N/A
Ethics oversight	N/A

Note that full information on the approval of the study protocol must also be provided in the manuscript.

Field-specific reporting

Please select the one below that is the best fit for your research. If you are not sure, read the appropriate sections before making your selection.

☒ Life sciences ☐ Behavioural & social sciences ☐ Ecological, evolutionary & environmental sciences

For a reference copy of the document with all sections, see nature.com/documents/nr-reporting-summary-flat.pdf

Life sciences study design

All studies must disclose on these points even when the disclosure is negative.

Sample size	Experimental sample sizes were chosen based on previous published studies from author and others, where statistically significant differences were detected for controls versus treated mice. There is no conclusion that is based on lack of difference between a control and an experimental group.<br>- Håglin S et al. J Neurosci 40: 4116-4129 (Naris occlusion).<br>- Maurya DK et al. Proc Natl Acad Sci 114:E9386-E9394 (2017). (Vismodegib)<br>- Norlin EM et al. J. Neurochem 93:1594-1602 (2005). (Odor)<br>- Otsuguro K et al. J Pharmacol Sci 97:510-518 (2005). (Forskolin)<br>Sample sizes used did take animal ethical guidelines into consideration.
Data exclusions	No data was excluded from analyses.
Replication	The number of independent biological replicates, i.e. mice are given for all experiments. For solely histological analyses, the replicates gave similar results. For experimental treatments, quantitative data is given in figures. Much preparatory effort (before collecting data for this study) was put on optimizing the different histological analyses, so that technical replicates yielded virtually the same result for a given sample when performed at different times .
Randomization	Randomization was not applied. Risks for covariations in control versus treated animal groups were reduced by performing experiments including control and treated mice on littermates and in parallell (both treatments and histological analyses). A considerable fraction (Fig. 1-6; Supplementary Fig. 1-4, 6-7) of the data is on tissues from control mice without treatments.
Blinding	For the measurements of stimulus-dependent disintegration of the MVT´s limiting membrane (Fig. 7 and 8), the experimenter was blind to experimental group allocation during data collection and analysis. For other experiments, blinding was not relevant as all biological replicates belonged to one group only (i.e. the rest of data is from control mice only).

Reporting for specific materials, systems and methods

We require information from authors about some types of materials, experimental systems and methods used in many studies. Here, indicate whether each material, system or method listed is relevant to your study. If you are not sure if a list item applies to your research, read the appropriate section before selecting a response.

Materials & experimental systems

n/a	Involved in the study
☐	☒ Antibodies
☒	☐ Eukaryotic cell lines
☒	☐ Palaeontology and archaeology
☐	☒ Animals and other organisms
☒	☐ Clinical data
☒	☐ Dual use research of concern

Methods

n/a	Involved in the study
☒	☐ ChIP-seq
☒	☐ Flow cytometry
☒	☐ MRI-based neuroimaging

Antibodies

Antibodies used

Guinea pig anti-M71/7236 (1:2,000, Lomvardas S et al. 2006),
Rabbit anti-RP2 (1:500, ProteinTech, 1415-1-AP),
Goat anti-CNGA2 (1:100, Santa Cruz Biotechnology, sc-13700),
Rabbit anti-AC3 (1:100, Santa Cruz Biotechnology, sc-588),
Rabbit anti-S100A5 (1:2000, Schäfer BW et al. 2000, 62FAC1),
Mouse anti-S100A5 (1:500, Thermo Fisher Scientific, MA5-29536, clone 4),
Rabbit anti-ARL3 (1:100, ProteinTech, 10961-1-AP),
Mouse anti-Synaptophysin (1:50, SySy, 101-011),
Rabbit anti-HGS/HRS (1:400, GeneTex, GTX101718),
Rabbit anti-STAM1 (1:100, 15 ProteinTech, 12434-1-AP),
Rat anti-LAMP1 (1:400, GeneTex, GTX42501, clone 1D4B),
Rat anti-LAMP2 16 (1:400, Thermo Fisher Scientific, 14-1072-82, clone ABL-93),
Mouse anti-Gβ (1:500, Santa Cruz 17 Biotechnology, sc-166123, H-1),
Mouse anti-GNAL (1:200, Santa Cruz Biotechnology, sc-55545, A-5),
Rat anti-TRPC2 (1:200, BiCell Scientific, 11012),
Rabbit anti-GNG13 (1:500, Atlas Antibodies, HPA046272),
Rat anti-CD9 (1:500, BD Biosciences, 553758, clone KMC8),
Rabbit anti-PDEδ (1:200, Atlas Antibodies, HPA037433),
Rabbit anti-β arrestin 2 (1:100, ProteinTech, 10171-1-AP),
Rabbit anti-Rab7 (1:400, ProteinTech, 55469-1-AP),
Rabbit anti-RTP (1:500, ProteinTech, 18973-1-AP),
Rabbit anti-AP1G1 (1:500, ProteinTech, 13258-1-AP),
Rabbit anti-LAMN2L (1:100, ProteinTech, 17877-1-AP),
Rabbit anti-CopII (1:500, Thermo Fisher Scientific, PA1-069A),
Rabbit anti-SEC31 (1:500, ProteinTech, 17913-1-AP),
Rabbit anti-GRASP55 (1:200, ProteinTech, 10598-1-AP),
Rabbit anti-GRASP65 (1:500, Thermo Fisher Scientific, PA3-910),
Rabbit anti-Rab10 (1:200, ProteinTech, 11808-1-AP),
Rabbit anti-Rab35 (1:200, Thermo Fisher Scientific, PA5-31674),
Rabbit anti-IFT20 (1:150, ProteinTech, 13615-1-AP),
Rabbit anti-IFT88 6 (1:150, ProteinTech, 13967-1-AP),
Rabbit anti-TSG101 (1:100, Novus Biologicals, NBP2-67884),
Rabbit anti-CHMP1A (1:500, ProteinTech, 15761-1-AP),
Rabbit anti-CHMP4B (1:200, Thermo Fisher Scientific, PA5-100092),
Rabbit anti-VPS4A (1:500, LifeSpan BioSciences, LS-C346226),
Rabbit anti-NSF (1:500, Novus Biologicals, NBP1-87035),
Alexa 488-conjugated anti-guinea pig (Jackson ImmunoResearch Europe, 706–545-148),
Alexa 488-conjugated donkey anti-rabbit (Life Technologies, A-21206),
Alexa 546-conjugated donkey anti-rabbit (Life Technologies, A-10040),
Alexa 546-conjugated donkey anti-goat (Life Technologies, A-11056),
Dylight 488-conjugated donkey anti-mouse (Agrisera AB, Sweden, AS101201),
Alexa 546-conjugated donkey anti-rat (Life Technologies, A-21208)
Cy™3 conjugated donkey anti-guinea pig (Jackson ImmunoResearch Europe, 706-165-148).

Validation

CNGA2 antibody (sc-13700) : validated in publication : doi.org/10.1002/dneu.22159 ,
AP1G1 antibody (13258-1-AP) : validated in publication : doi.org/10.3389/fcell.2019.00181 ,
ARL3 antibody (10961-1-AP) :validated in publication : doi.org/10.1038/ncomms6295 ,
Beta Arrestin 2 antibody (10171-1-AP) : validated in publication : doi.org/10.1038/cddis.2016.89 ,
CHMP1A antibody (15761-1-AP) : validated in publication : doi.org/10.1016/j.celrep.2018.06.100 ,
CHMP4B antibody (PA5-100092) : validated by manufactures using blocking peptides : https://www.thermofisher.com/antibody/product/CHMP4B-Antibody-Polyclonal/PA5-100092 ,
COPII antibody (PA1-069A) : validated in publication : doi.org/10.1016/j.jbc.2021.101536 ,
GRASP55 antibody (10598-1-AP) : validated in publication : doi.org/10.4049/jimmunol.1901124 doi.org/10.1083/jcb.200907132 ,
GNG13 antibody (HPA046272) : validated in publication : doi.org/10.1016/j.prp.2020.153143 doi.org/10.1038/s41586-018-0393-7 ,
GRASP65 antibody (PA3-910): validated in publication : doi.org/10.1038/s41598-019-53124-2 doi.org/10.1007/s10495-019-01579-z ,
HGS antibody (GTX101718) : validated in publication : doi.org/10.1371/journal.ppat.1005123 ,

IFT20 antibody (13615-1-AP) : validated in publication : doi.org/10.1111/cas.13970 and doi.org/10.1038/s41418-019-0357-y ,
IFT88 antibody (13967-1-AP) : validated in publication : doi.org/10.1038/s41388-018-0211-6 ,
LMAN2L antibody (17877-1-AP) : Manufacturer's confirmation with siRNA : https://www.ptglab.com/products/LMAN2L-Antibody-17877-1-AP.htm ,
LAMP1 antibody (GTX42501) : validated in publication : doi.org/10.1016/S0021-9258(18)34878-6 ,
LAMP2 antibody (14-1072-82) : validated in publication : doi.org/10.1016/j.bbrep.2016.01.010 ,
NSF antibody (NBP1-87035) : validated in publication : doi.org/10.1007/s12031-014-0231-9 ,
S100A5 antibody (MA5-29536) : validated in author´s laboratory by unilateral naris occlusion
Rab10 antibody (11808-1-AP) : validated in publication : doi.org/10.1074/jbc.RA118.007318 ,
Rab35 antibody (PA5-31674) : validated by manufactures using blocking peptides : https://www.thermofisher.com/antibody/product/RAB35-Antibody-Polyclonal/PA5-31674 ,
Rab7 antibody (55469-1-AP) : validated in publication : doi.org/10.1038/s41419-021-03670-3 ,
RP2 antibody (14151-1-AP) : validated in publication : doi.org/10.1016/j.stemcr.2020.05.007 ,
RTP1 antibody (18973-1-AP) : validated by manufacturer : https://www.thermofisher.com/antibody/product/RTP1-Antibody-Polyclonal/18973-1-AP ,
SEC31 antibody (17913-1-AP) : validated in publication : doi.org/10.1038/s41586-021-04109-7 ,
Synaptophysin antibody (101 011) : validated in publication : doi.org/10.1046/j.1460-9568.1999.00542.x ,
STAM1 antibody (12434-1-AP) : validated in publication : doi.org/10.1074/jbc.M116.757138 ,
TSG101 antibody (NBP2-67884) : validated in publication : doi.org/10.1111/jcmm.16002 ,
VPS4A antibody (LS-C346226) : manufacturer's confirmation: using WB and IHC: https://www.lsbio.com/antibodies/vps4a-antibody-if-immunofluorescence-ihc-wb-western-ls-c346226/357176 ,
Golfα (GNAL) antibody (sc-55545) : validated in publication : doi.org/10.1038/s41467-017-02661-3 and doi.org/10.1093/schbul/sbv129 ,
TRPC2 antibody (11012) : manufacturer's confirmation by IHC and confirmation in author´s laboratory: https://bicellscientific.com/product/trpc2-antibody/ ,
M71/M72 antibody : validated in publication : Lomvardas S et al. 2006 ,
AC3 antibody (sc-588) : validated in publication : doi.org/10.1016/S0896-6273(00)00060-X ,
S100A5 antibody (62FAC1) : validated in publication : Schäfer BW et al. 2000 ,
Gβ1 antibody (sc-166123) : validated in publication : doi.org/10.7554/eLife.54298 and doi.org/10.3892/etm.2022.11450 ,
CD9 antibody (553758) : validated in publication : doi.org/10.4049/jimmunol.166.5.3256 ,
PDEδ antibody (HPA037433) : manufacturer's confirmation: using WB and IHC : https://www.atlasantibodies.com/products/antibodies/primary-antibodies/triple-a-polyclonals/pde6d-antibody-hpa037433 ,

Animals and other research organisms

Policy information about studies involving animals; ARRIVE guidelines recommended for reporting animal research, and Sex and Gender in Research

Laboratory animals	C57Bl/6J mice (8-12 weeks old) obtained from colonies established in-house were kept in IVCs (< 5/cage) on a 12 h light/dark cycle, 21 (± 1) °C and 55 (± 5) % humidity, at the Umeå Center for Comparative Biology, Umeå, Sweden.
Wild animals	Not applicable
Reporting on sex	Mice of both sexes were used in this study.
Field-collected samples	Not applicable
Ethics oversight	All animal experiments were conducted according to the ethical approval by the Local Ethics Committee for Animal Research at the Court of Appeal for the upper northern area of Norrland (Umeå, Sweden).

Note that full information on the approval of the study protocol must also be provided in the manuscript.
